# Ginseng

**Published:** 1885-06

**Authors:** 


### Ginseng.

A parliamentary paper contains the account of a journey made by the Consul-General of Great Britain in Corea. Some interesting information is given with regard to the production of the famous drug ginseng, so prized as a tonic by the Chinese. It is grown from a seed which is sown in March. The seedlings are planted out in beds raised a foot above the level of the surrounding soil, bordered with upright stakes, and covered in from sun and rain by sheds of reeds, well closed in except toward the north side, where they are left open. In the first or second year the ginseng plant is only two or three inches high, and has only two leaves. It is transplanted frequently during this period. In the fourth year the stem is about six inches high, with four horizontal leaves standing out from it at right angles, and in the fifth year a strong, healthy plant has reached maturity, though it is more usual not to take it up until it has reached the sixth season. Ordinary ginseng is prepared by simply drying the root in the sun or over a charcoal fire. To make red or clarified ginseng, the root is placed in wicker baskets, which are put in a large earthenware vessel with a closely fitting cover, and pierced at the bottom with holes. It is then placed over boiling water, and steamed for about four hours.

Ginseng was for centuries regarded as a very elixir of life all over the East; and especially in China and Japan. Its properties were supposed to be miraculous, but they were generally supposed to be confined to the Corean ginseng. But its enormous price put it out of the reach of the poorer classes. The wild ginseng of Corea has frequently fetched twenty times its weight in silver in China. The export from Corea is a strict monopoly, which affords a considerable revenue, and is said to be the king's personal perquisite. Death is the punishment for smuggling it out of the country. The total export is only about 27,000 pounds avoirdupois.—*Scientific American.*

